# Functionalization of Radiolabeled Antibodies to Enhance Peripheral Clearance for High Contrast Brain Imaging

**DOI:** 10.1021/acs.molpharmaceut.2c00536

**Published:** 2022-10-06

**Authors:** Eva Schlein, Stina Syvänen, Johanna Rokka, Tobias Gustavsson, Raffaella Rossin, Marc Robillard, Jonas Eriksson, Dag Sehlin

**Affiliations:** † Department of Public Health and Caring Sciences, Uppsala University, 751 85 Uppsala, Sweden; ‡ Tagworks Pharmaceuticals, Toernooiveld 1, 6525 ED Nijmegen, Netherlands; § PET Centre, Uppsala University Hospital, 751 85 Uppsala, Sweden; ∥ Department of Medicinal Chemistry, Uppsala University, 751 23 Uppsala, Sweden

**Keywords:** clearing agent, mannose, TCO, tetrazine, inverse electron-demand Diels−Alder reaction, Alzheimer’s disease, SPECT imaging, antibody

## Abstract

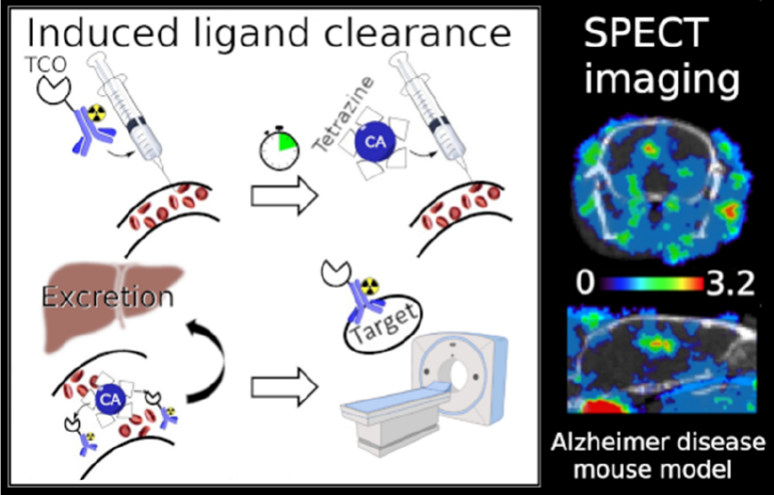

Small molecule imaging agents such as [^11^C]PiB, which bind to the core of insoluble amyloid-β (Aβ) fibrils, are useful tools in Alzheimer’s disease (AD) research, diagnostics, and drug development. However, the [^11^C]PiB PET signal saturates early in the disease progression and does not detect soluble or diffuse Aβ pathology which are believed to play important roles in the disease progression. Antibodies, modified into a bispecific format to enter the brain via receptor-mediated transcytosis, could be a suitable alternative because of their diversity and high specificity for their target. However, the circulation time of these antibodies is long, resulting in an extended exposure to radiation and low imaging contrast. Here, we explore two alternative strategies to enhance imaging contrast by increasing clearance of the antibody ligand from blood. The bispecific Aβ targeting antibody RmAb158-scFv8D3 and the monospecific RmAb158 were radiolabeled and functionalized with either α-d-mannopyranosylphenyl isothiocyanate (mannose) or with *trans*-cyclooctene (TCO). While mannose can directly mediate antibody clearance via the liver, TCO-modified antibody clearance was induced by injection of a tetrazine-functionalized, liver-targeting clearing agent (CA). In vivo experiments in wild type and AD transgenic mice demonstrated the ability of both strategies to drastically shorten the circulation time of RmAb158, while they had limited effect on the bispecific variant RmAb158-8D3. Furthermore, single photon emission computed tomography imaging with TCO-[^125^I]I-RmAb158 in AD mice showed higher contrast 1 day after injection of the tetrazine-functionalized CA. In conclusion, strategies to enhance the clearance of antibody-based imaging ligands could allow imaging at earlier time points and thereby open the possibility to combine antibodies with short-lived radionuclides such as fluorine-18.

## Introduction

The introduction of the positron emission tomography (PET) ligand [^11^C]Pittsburg compound B ([^11^C]PiB) about 2 decades ago enabled new possibilities to study and diagnose Alzheimer’s disease (AD), as it binds and visualizes amyloid-beta (Aβ) plaques in the living brain.^1^ However, further investigations showed that brain retention of the radiotracer remained static during disease progression and that Aβ pathology in carriers of a specific APP mutation as well as AD patients with predominantly diffuse Aβ plaques cannot be visualized with this method.^2−4^ Furthermore, the soluble forms of Aβ, which are not visualized with [^11^C]PiB, correlate with neurotoxicity better than Aβ plaques.^5^ To image soluble and diffuse aggregates of Aβ, antibody-based imaging techniques could be a suitable approach and an alternative to amyloid radioligands such as [^11^C]PiB.

**Figure 1:**
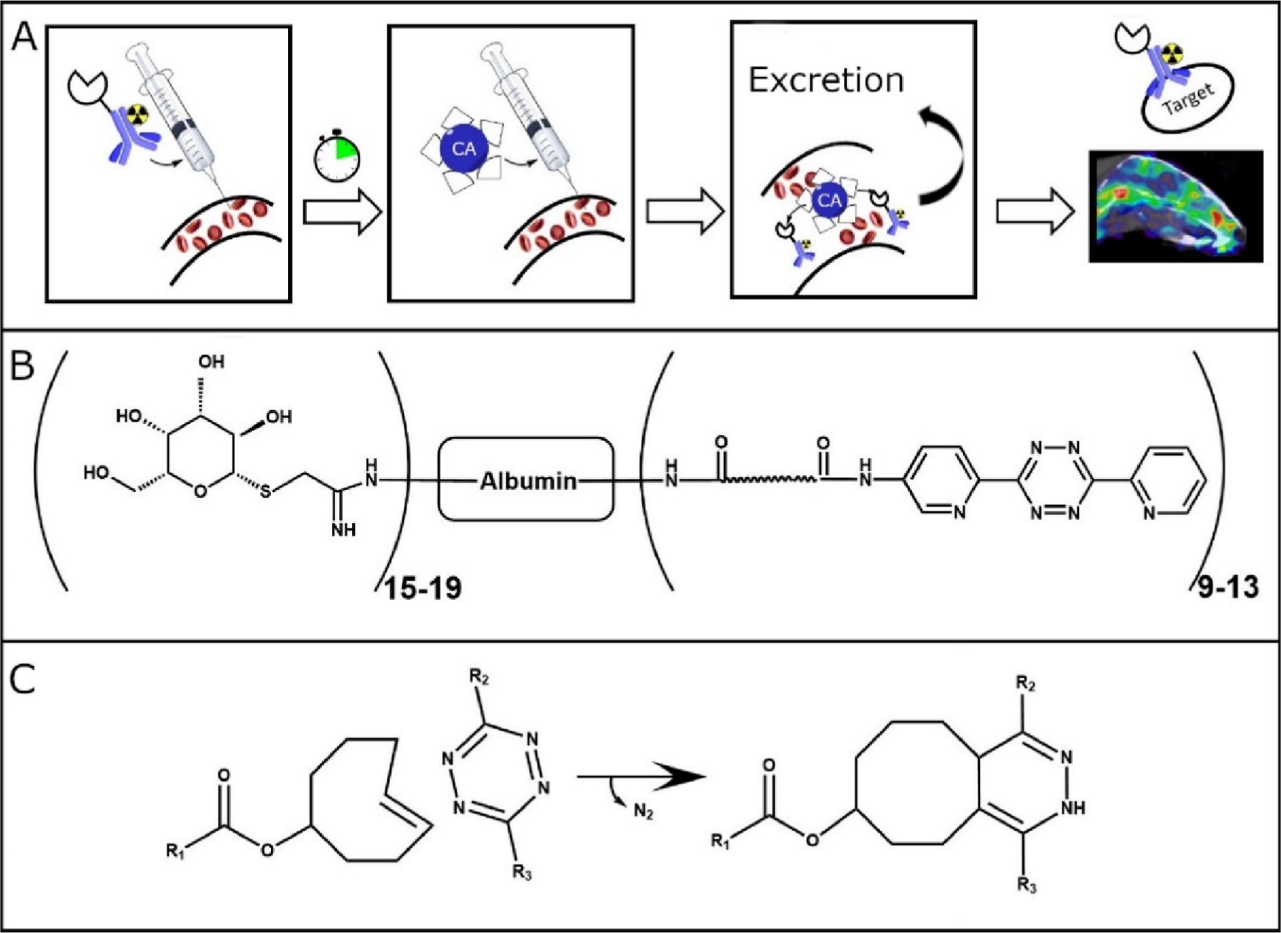
(A) Schematic description of the principle for a CA. (B) Schematic representation of a tetrazine-functionalized CA (galactose–albumin–tetrazine). (C) General principle of the IEDDA reaction; TCO reacts with tetrazine and generates nitrogen as a side-product when a covalent bond is formed.

Antibody-based imaging techniques have been in focus of research for a while. Despite the high specificity and selectivity, the size of the antibody is often a drawback for its use as an imaging ligand. The majority of antibody ligands are IgG-based^6^ and, due to the Fc receptor,^7^ the biological half-life of the ligand is long. This long circulation time results in poor contrast, high background signals, and, due to the usage of long-lived radioisotopes, unwanted radiation exposure to normal tissue.^8−10^ To counteract this issue, clearing agents (CAs) can be a useful approach (Figure 1A). The task of a CA is to remove radiolabeled antibody from the circulation to enhance the target-to-blood ratio and thereby increase the image quality. Early CAs were based on antibodies raised against the injected antibody ligand, to lower the blood levels of the ligand,^11^ or used the avidin–biotin interaction.^12^ However, these methods were prone to side-effects, such as immunogenic responses.^13^

An interesting approach to increase antibody clearance, with low side-effects, is the addition of a hexose, such as mannose, to the radioimmunoconjugate. Mannose-modified proteins have been shown to be cleared fast from the circulation by macrophage mannose receptors, which can be found on various tissues, such as endothelial cells in the liver.^14−16^ Bio-orthogonal reactions are a useful approach to efficiently clear antibodies with no known side-effects.^17,18^ A previously developed CA (Figure 1B) has shown promising results in tumor imaging.^19^ This CA is modified with galactose, to actively target the liver via Ashwell receptors on hepatocytes,^19^ and functionalized with a tetrazine, which allows in vivo interaction with a *trans*-cyclooctene (TCO)-functionalized antibody through the inverse-electron demand Diels–Alder (IEDDA) reaction (Figure 1C). In this reaction, an electron-deficient diene (tetrazine) and an electron-rich dienophile (TCO) react upon eliminating nitrogen to form a stable bond. This is an irreversible reaction, which is catalyst-free, selective, and mild, as well as compatible with biological systems.^20^ Both of the reaction partners, that is, the tetrazine and the TCO, can be introduced into different biomolecules via enzymes or chemical modifications^21^ and various applications of the reaction have been explored.^20,22,23^

We have previously developed an antibody,^24^ mAb158, which specifically targets Aβ protofibrils, a soluble form of Aβ aggregates.^24^ Further development resulted in a bispecific recombinant antibody, RmAb158-scFv8D3, where mAb158 is fused to a single chain variable fragment (scFv) of a rat anti-mouse transferrin receptor (TfR) antibody (8D3), enabling the antibody to cross the blood–brain-barrier up to 80-times more than unmodified antibody.^25^ These antibodies have been used for PET or single photon emission computed tomography (SPECT) imaging in transgenic mice with Aβ pathology.^25−30^ Both recombinant, IgG-based antibodies, RmAb158-scFv8D3 and RmAb158, were included in this study.

The aim of this study was to investigate the usage of two different clearing approaches (mannose modification and CA) on the two above-mentioned antibodies to achieve an increase in contrast for brain-imaging purposes.

## Materials and Methods

### Antibody Production

RmAb158-scFv8D3^31^ and RmAb158,^24^ which both bind selectively to Aβ protofibrils, were expressed as previously described.^32^ In short, Expi293F mammalian cells were transiently transfected with pcDNA3.4 vectors encoding the sequences for the heavy and light chains of the antibody variants. The antibody was purified and separated from the cell medium with an ÄKTA system (5 mL HiTrap Protein G HP, 17-0405-01, GE Healthcare) and the buffer was subsequently exchanged to phosphate-buffered saline (PBS; pH 7.4).

### Antibody Modifications

**Figure 2:**
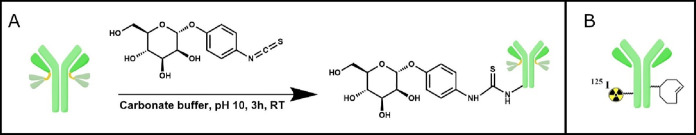
(A) Reaction scheme of a bispecific antibody mannose modification. (B) Schematic overview of a double-functionalized monospecific antibody.

RmAb158-scFv8D3 and RmAb158 were mannosylated with α-d-mannopyranosylphenyl isothiocyanate (Figure 2A). Isothiocyanates (SCN) contain an electrophilic carbon atom, which can react with nucleophiles, such as hydroxyl, amino, or thiol groups (such as tyrosine, lysine, or cysteine) to form O-thiocarbamates, thiourea, or dithiocarbamate. The antibody was dissolved in PBS to 1 mg/mL and 0.1 M sodium carbonate buffer, pH 9.5, was added to a final concentration of 34 mM. A 20-fold molar excess of α-d-mannopyranosylphenyl isothiocyanate (Sigma-Aldrich, Stockholm, Sweden) to antibody was added and the preparation was incubated for 3 h at room temperature (RT). Unreacted α-d-mannopyranosylphenyl isothiocyanate was removed with Zeba spin desalting columns (7K MWCO, 0.5 mL, 89882, Thermo Fisher, Uppsala, Sweden) and eluted in PBS.

Antibodies aimed for induced clearance by tetrazine-functionalized CA were modified with TCO-groups. To achieve this type of modification, the amino-group on lysine residues of the antibody were functionalized with an NHS-activated TCO-tag (Figure 2B). The antibody, typically 1 mg/mL in PBS, supplemented with 30 mM sodium carbonate buffer (1:1 mix of 1 M Na_2_CO_3_ and 1 M NaHCO_3_, pH 8.0) was reacted with TCO–NHS^33^ dissolved in dimethyl sulfoxide to 10 mM at a 20-fold TCO–antibody molar ratio, which typically results in approximately 3 TCO groups per antibody molecule.^34^ The solution was incubated for 3 h in darkness while shaking at 600 rpm and subsequently purified from remaining unreacted TCO with Zeba spin desalting columns (7K MWCO, 0.5 mL, Thermo Fisher) and eluted in PBS.

To verify the functionality of the TCO modification, TCO-modified RmAb158-scFv8D3 or RmAb158 was mixed with tetrazine-functionalized CA (3.0 mg/mL) and incubated for 30 min while shaking at 600 rpm. Bolt 4× LDS sample buffer (B0007, Thermo Fisher) was added to the sample that was subsequently loaded on a 12 well Bolt 8% Bis–Tris sodium dodecyl sulfate-polyacrylamide gel electrophoresis (SDS-PAGE) gel (NW04122BOX, Thermo Fisher), which was run at 200 V for 35 min. If the samples were radioactive, the PAGE-gel was exposed to a phosphorminaging plate overnight and the next day scanned with a Cyclone Plus Imager system (PerkinElmer) at a resolution of 600 dpi. The gel was stained for proteins with PageBlue staining solution (24620, Thermo Fisher).

### Radiolabeling

We selected iodine-125 for radiolabeling due to its non-residualizing properties that facilitate rapid elimination of potentially free iodine to assure that the majority of the detected radioactivity was derived from intact antibody.^42^ For radioiodination of antibodies, the chloramine-T method was used^35^ in which chloramine-T oxidizes iodine-125 to its cationic form and reacts with the anionic form of tyrosine to form [^125^I]I-tyrosine.^36^ On average 102 ± 36.37 μg of antibody was labeled with 42.3 ± 24.91 MBq iodine-125 (PerkinElmer Inc., Waltham, MA, USA) depending on the experimental setup. Chloramine-T (Sigma Aldrich) was added (5 μg, 200 μM in PBS) and incubated for 90 s at RT. The reaction was quenched by the addition of sodium-metabisulfite (10 μg, 440 μM in PBS, Sigma-Aldrich). After purifying the radiolabeled antibody with disposable Zeba spin desalting columns (7K MWCO, 0.5 mL, 89882, Thermo Fisher), the final activity was measured in an ion chamber. For experiments with radiolabeled TCO-modified antibody (Figure 2B), radiolabeling was performed before TCO-modification to prevent damage of TCO induced by chloramine-T.^37^

### ELISA Evaluation of Antibody Ligands

To investigate if mannose, TCO, or iodine-125 functionalization of the antibody ligands affected their functionality, TfR and Aβ ELISA binding assays, as well as an anti-mouse IgG sandwich-ELISA for antibody quantification, were performed as previously described.^25^ In brief, 96-well half area plates (Corning) were coated with TfR (1 μg/mL; Sino Biological, Beijing, China), Aβ (100 nM; Innovagen, Lund, Sweden), or anti-mouse (0.5 μg/mL, Vector, Oxfordshire, UK) and blocked with BSA (1% in PBS), followed by addition of serial dilutions of the antibody ligands. Bound ligand was detected with HRP-conjugated anti-mouse-IgG-F(ab′)_2_ (Jackson ImmunoResearch Laboratories, West Grove, PA, USA), and signals were developed with K-Blue Aqueous TMB substrate (Neogen Corp., Lexington, KY, USA) and read with a spectrophotometer at 450 nm. All dilutions were made in ELISA incubation buffer (PBS, 0.1% BSA, 0.05% Tween-20).

### Animal Experiments

All experiments were performed in wild-type (wt) or transgenic (tg-ArcSwe) mice. Tg-ArcSwe is a mouse model of Aβ pathology,^38^ which harbors the Swedish and Arctic amyloid precursor protein mutations that cause early onset of AD. Tg-ArcSwe mice were studied at 18–20 months of age, that is, when the Aβ pathology has reached an advanced stage, and compared with wt mice of the same age. Genotype, age, and amount of injected radioactivity for each of the experiments are presented in Table 1.

**Table 1:** Study Summary with Number of Animals

experiment	animals *n* (wt/tg)	age (months)	injected activity (MBq)	injected dose (nmol/kg)
[^125^I]I-RmAb158	(-/3)	18–20	0.25 ± 0.03	0.62 ± 0.01
[^125^I]I-RmAb158-scFv8D3	(-/3)	18–20	0.27 ± 0.04	0.44 ± 0.01
mannose-[^125^I]I-RmAb158	(5/-)	4–8	0.54 ± 0.02	0.53 ± 0.04
mannose-[^125^I]I-RmAb158-scFv8D3	(9/-)	4–8	0.36 ± 0.04	0.32 ± 0.07
TCO-RmAb158 ± CA	(5/-)	4–8	n.a.	3.67 ± 0.07
TCO-RmAb158-scFv8D3 ± CA	(6/-)	4–8	n.a.	2.51 ± 0.06
TCO-[^125^I]I-RmAb158 + CA	(-/6)	18–20	1.13 ± 0.06	2.22 ± 0.58
TCO-[^125^I]I-RmAb158 + CA	(6/-)	3	1.01 ± 0.08	1.31 ± 0.19
TCO-[^125^I]I-RmAb158 + CA + SPECT imaging	(3/5)	18–20	9.07 ± 0.23	4.12 ± 0.21

All procedures described in this study were approved by the Uppsala County Animal Ethics board (C17/14 and 5.8.18-13350/2017) and were in accord with the rules and regulations of the Swedish Animal Welfare Agency and complied with the European Communities Council Directive of 22 September 2010 (2010/63/EU).

### Ex Vivo and Pharmacokinetic Studies

Wt or tg-ArcSwe mice were injected with the antibodies RmAb158 or RmAb158-scF8D3 either non-modified or functionalized with mannose or TCO, with or without radiolabel, according to Table 1. Blood or plasma samples were taken from the tail vein, typically at 1, 2, 3, 4, 6, 24, 48, and 72 h after injection. For experiments with tetrazine-functionalized CA, each mouse received an intravenous injection of the CA (10 mg/kg body weight) 1 or 72 h after TCO–antibody administration in the initial pharmacokinetic studies or 72 h after antibody injection in SPECT studies. The CA dose was based on previous experience^19^ to obtain a large molar excess of tetrazine over TCO and efficient clearance of antibody from the blood. Whole blood or plasma samples were taken before CA administration and at 5 min, 15 min, 30 min, 1 h, and 2 h after CA administration. In all studies, terminal blood and plasma samples were obtained by heart puncture at 24, 72, or 96 h after antibody injection, prior to intracardiac perfusion with 0.9% saline solution for 3 min.

Quantification of non-radiolabeled antibody was performed in plasma with anti-Aβ ELISA, following the same protocol as described above for evaluation of antibodies. A standard series of known antibody concentrations was used for quantification. From mice injected with radiolabeled antibody, brain, cerebellum, and peripheral organs (lung, liver, kidney, spleen, heart, muscle, bone, pancreas, and skull) were isolated. Antibody concentrations in blood and organs were quantified by radioactivity measurements using a gamma counter (1480 Wizard, Wallac Oy, Turku, Finland). Activity concentration was corrected for radioactive decay to the time of injection and presented as percent of injected dose per gram tissue (% ID/g) or as an organ-to-blood concentration ratio.

Extracted brains were immediately frozen at −80 °C after perfusion, sectioned on a cryostat (20 μm), and mounted on Superfrost Plus slides (Menzel GmbH, Braunschweig, Germany). After cryosectioning, brain slides were exposed to phosphor imaging plates (MS, MultiSensitive, PerkinElmer, Downers Grove, IL, USA) overnight. Plates were scanned in a Cyclone Plus phosphor imager (PerkinElmer) at 600 dpi.

For ex vivo assessment of antibody–CA interaction, radiolabeled TCO-modified antibody, as well as plasma samples from mice injected with TCO-modified antibody were incubated with or without CA for 30 min at RT and subsequently mixed with Bolt LDS sample buffer and applied on a 8% NuPAGE Bis–Tris gel and run with MOPS buffer at 200 V for 35 min. Depending on amount of radioactivity, the gels were exposed for 5–15 min up to overnight and stained with PageBlue Protein Staining Solution afterward. The stained gels were captured with ChemiDoc XRS+ (BioRad, Hercules, CA, USA).

### Immunofluorescence Staining and Nuclear Track Emulsion Autoradiography

For CD31 staining, cryosections of 20 μm were fixed in cold methanol for 10 min and blocked for 1 h with normal goat serum, followed by tissue permeabilization in 0.1% Tween 20 in PBS for 5 min. The sections were incubated overnight with rat anti-mouse CD31 (BD Biosciences, Catalog no. 553370) at 4 °C subsequently and incubated with Alexa-488 goat-anti-rat IgG for 1 h at RT.

For Aβ staining, cryosections of 20 μm were fixed in 4% formaldehyde for 20 min and washed in PBS, followed by antigen retrieval with 25 mM citric acid buffer (pH 7.3) and subsequent dipping in 70% formic acid for 5 min ad RT. Unspecific binding was blocked with a M.O.M IgG basic kit (Vector, Catalog no. BMK 2202). For subsequent permeabilization, the sections were incubated with 0.4% Triton X-100 in PBS for 5 min. After incubation with the M.O.M diluent (200 μL of concentrated protein stock solution in 2 mL of PBS with 0.4% TritonX-100) for 5 min, the primary antibody (6E10, Nordic Biosite) was diluted in M.O.M diluent and incubated overnight at 4 °C on a shaker. Alexa-594 secondary goat-anti-mouse antibody (Thermo Fisher, Stockholm, Sweden) in 0.1% Tween-20 was applied for 1 h at RT.

Nuclear track emulsion autoradiography was performed in darkness following the previously described protocol.^25,28,39^ In short, immunostained sections were submerged in ILFORD K5 emulsion, air-dried for 2 h at RT and exposed for 4 weeks at 4 °C. The emulsion-covered tissue sections were developed according to the manufacturer’s manual, dehydrated in an increased series of ethanol solution and mounted with Pertex mounting medium. After development, immunofluorescence and emulsion staining were imaged with a Zeiss Observer Z.1 microscope using the ZEN 2.6 software (Carl Zeiss Microimaging GmbH, Jena, Germany).

### SPECT Imaging

A subset of mice injected with TCO-[^125^I]I-RmAb158 were investigated with SPECT imaging, before and after CA administration. SPECT scans were performed 3 or 5 days after injection of radiolabeled antibody, as well as 2 and 24 h after CA administration. Each mouse was scanned maximum three times. Mice were anesthetized with 4% sevoflurane before scanning, positioned on the pre-heated scanner bed of a small animal nanoScan SPECT/CT (Mediso Medical Imaging Systems, Hungary) and scanned for 45–90 min depending on the selected field of view (whole body or head scan). CT was performed with the following settings: 50 kilovoltage peak X-ray, 600 μA, and 480 projections; and CT images were reconstructed using filtered back projections. Iodine-125 γ-emission was collected with an acquisition frame of 2 min. SPECT acquisition data were reconstructed using Nuclide 2.03 software and Tera-TomoTM 3D SPECT reconstructive algorithm (Mediso Medical Imaging Systems, Hungary) with scattering and attenuation correction. SPECT images were reconstructed using 48 iterations into a static image. Reconstructed data were decay-corrected and adjusted for injected dose. Data were visualized in AMIDE v 1.0.4.^40^

### Data Analysis

Values are reported as mean ± standard deviation. Data were analyzed using two-way analysis of variance (ANOVA) followed by Bonferroni’s *post hoc* test or unpaired, parametric *t*-test. Analysis was performed using the Prism 9 software (GraphPad Software, Inc., La Jolla, CA, USA).

## Results

### Mannosylation

**Figure 3:**
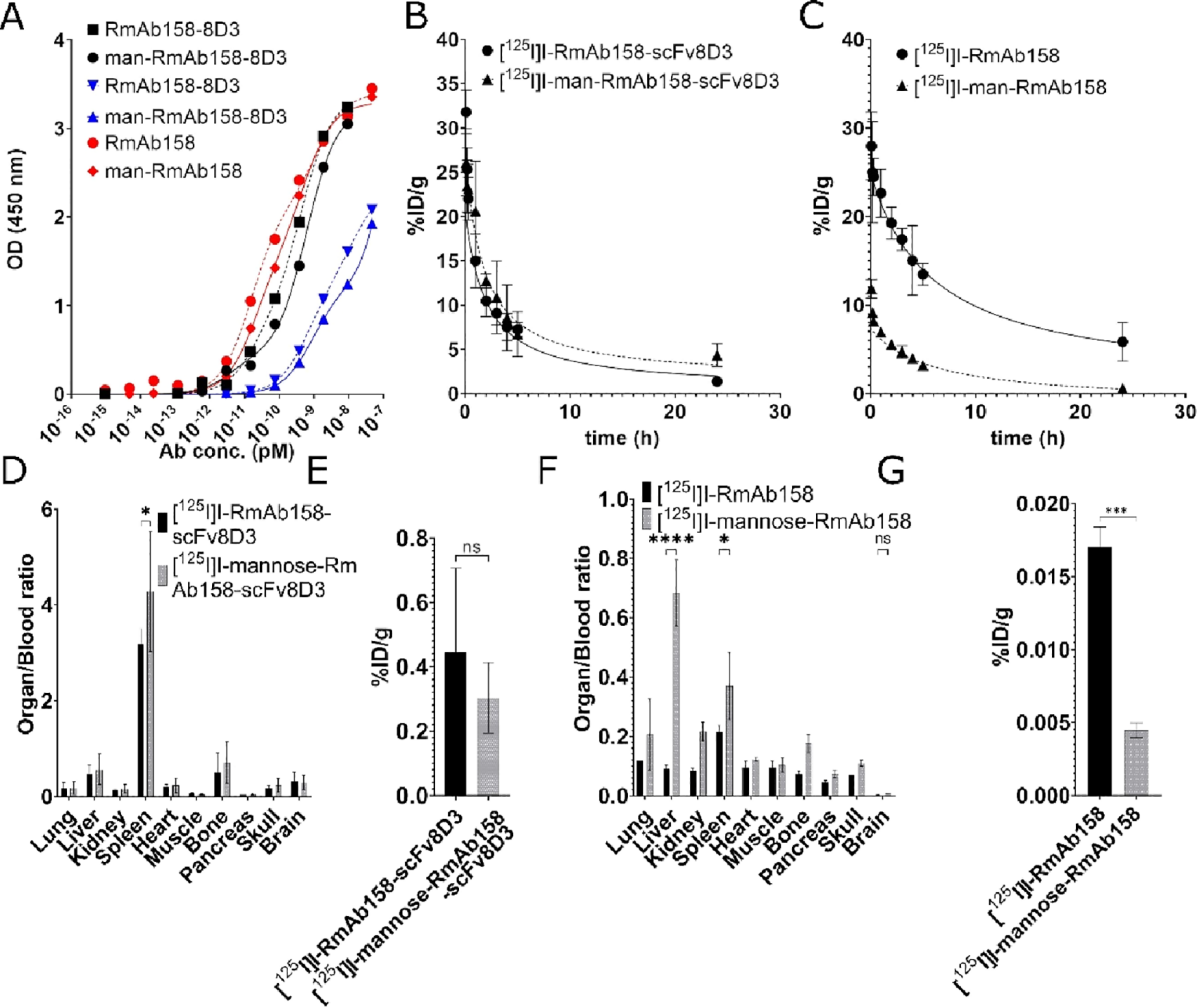
Mannose modification of RmAb158-scFv8D3 and RmAb158. (A) Aβ affinity ELISA analysis of RmAb158-8D3 (black) and RmAb158 (red) and TfR affinity ELISA analysis of RmAb158-8D3 (blue), with and without mannose modification of the antibodies. (B) [^125^I]I-RmAb158-scFv8D3 (0–2 h, *n* = 5; 0–24 h, *n* = 2) and mannose-[^125^I]I-RmAb158-scFv8D3 (*n* = 3) blood concentration over time (C). [^125^I]I-RmAb158 (*n* = 2) and mannose-[^125^I]I-RmAb158 (*n* = 3) blood concentration over time (D). [^125^I]I-RmAb158-scFv8D3 and mannose-[^125^I]I-RmAb158-scFv8D3 biodistribution 24 h after injection, expressed as an organ-to-blood ratio (E). Mannose-[^125^I]I-RmAb158-scFv8D3 brain distribution 24 h after injection (F). Mannose-[^125^I]I-RmAb158 biodistribution 24 h after injection, expressed as an organ-to-blood-ratio (G). Mannose-[^125^I]I-RmAb158 brain distribution 24 h after injection. Data are represented in mean ± standard deviation. Significant differences between groups were tested with two-way ANOVA, followed by Bonferroni’s *post hoc* test (D,F), or unpaired *t*-test (E,G) (*p < 0.05, ***p < 0.001, ****p < 0.0001; ns = non-significant).

The antibodies RmAb158-scFv8D3 and RmAb158 were mannosylated to assess the effect of direct modification for increased antibody clearance. To investigate if the binding affinity of the antibodies was affected by the modification, Aβ and TfR ELISAs were performed (Figure 3A). Compared to unmodified antibody, the affinity of mannosylated antibodies to their targets was not influenced.

Radiolabeled, mannosylated, and non-modified antibody were injected in wt mice, and blood samples were taken at different time points to study how mannose-modification affected antibody clearance from blood. For mannose-[^125^I]I-RmAb158-scFv8D3, the blood concentration 5 min post injection was on average 26% ID/g and then decreased to around 4% ID/g at 24 h. In comparison, [^125^I]I-RmAb158-scFv8D3 showed an initial average blood concentration of 32% ID/g, which then decreased to 1.4% ID/g at 24 h post injection (Figure 3B). Thus, mannose modification had no major impact on RmAb158-scFv8D3 and there was even a trend indicating that the non-mannosylated bispecific antibody was cleared faster than the mannosylated version.

For [^125^I]I-RmAb158, there was a clear effect of mannosylation already 5 min after injection. Initial concentration of mannose-[^125^I]I-RmAb158 was on average 12% ID/g and then decreased to 0.6% ID/g, compared to [^125^I]I-RmAb158, which displayed 27% ID/g and 6% ID/g at 5 min and 24 h, respectively (Figure 3C). This indicated that mannose modified RmAb158 was cleared from the blood substantially faster than unmodified RmAb158 and that clearance was efficient already at the earliest studied time point.

To understand how the antibody was distributed in the body in relation to blood, organ-to-blood ratios were calculated. [^125^I]I-RmAb158-scFv8D3 was to a large degree distributed to the spleen, as previously reported,^31^ but there were no major differences between mannosylated and unmodified bispecific antibody in distribution to peripheral organs (Figure 3D) or brain (Figure 3E).

[^125^I]I-RmAb158 without modification showed an even distribution in the organs, with only a slightly higher relative concentration in the spleen compared to the other organs. In contrast, after injection of [^125^I]I-RmAb158 modified with mannose, a high relative concentration was recorded in the liver as well as in the spleen, with some increase also in the kidney and bone, either as a direct or indirect consequence of mannose mediated uptake and degradation in the liver (Figure 3F). Notably, as a consequence of lower exposure of mannose-[^125^I]I-RmAb158 over the course of the experiment, its absolute brain concentration was substantially decreased compared to unmodified [^125^I]I-RmAb158 (Figure 3G). Mannose-induced antibody clearance as a means to increase imaging contrast was therefore not further pursued.

### Tetrazine-Functionalized CA

**Figure 4:**
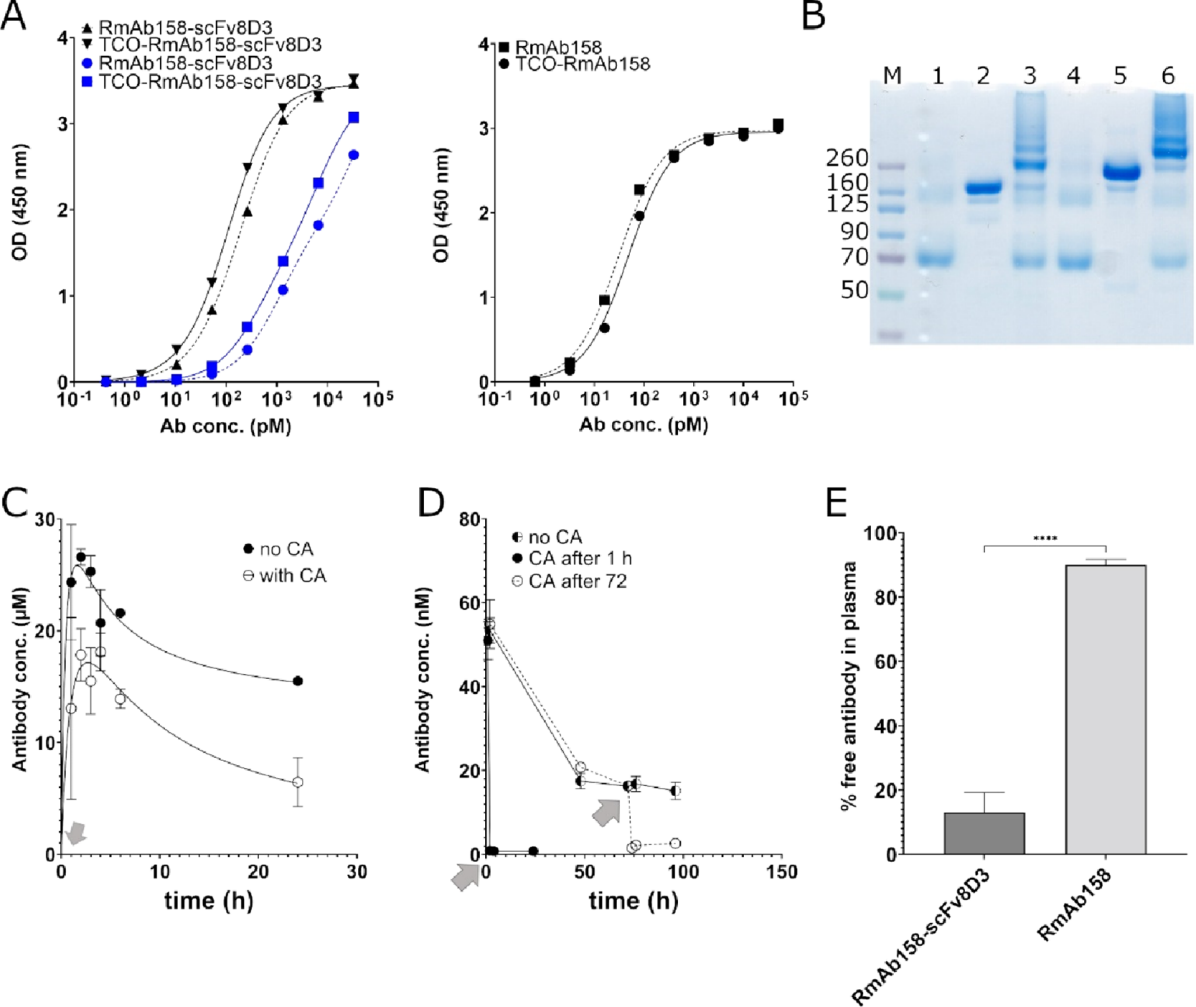
Tetrazine-functionalized CA. (A) ELISA analysis of modified antibodies binding to Aβ protofibrils (black) and TfR (blue) before and after TCO-modification. (B) SDS-PAGE showing CA (1 and 4), and the antibodies TCO-RmAb158 and TCO-RmAb158-scFv8D3 before (2 and 5) and after (3 and 6) click reaction with CA. (C) Plasma concentration of TCO-RmAb158-scFv8D3 with administration of CA 1 h after antibody injection (indicated by gray arrow). (D) Plasma concentration of TCO-RmAb158 with CA administered at 1 or 72 h after antibody injection (indicated by gray arrows). (E) Free plasma conc. of [^125^I]I-RmAb158-scFv8D3 and [^125^I]I-RmAb158 2 h after injection. Data are represented in mean ± standard deviation. Significant differences between groups were tested with unpaired *t*-test (E) (****p < 0.0001).

To investigate whether antibody clearance could be induced at a given time point by injection of a CA, similar analyses were done for TCO-modified variants of RmAb158-scFv8D3 and RmAb158. This approach takes advantage of an initial distribution phase, where the antibody can accumulate at its target—in this case the brain—before clearance is induced by administration of a large excess of tetrazine-functionalized CA. First, TCO-modified antibodies were tested for their functionality with ELISA and SDS-PAGE (Figure 4A,B). ELISA data showed that the TCO-functionalized antibody did not differ in Aβ or TfR binding, compared with the unmodified control (Figure 4A). SDS-PAGE analysis showed that TCO modified antibodies could react in vitro with the CA. After conjugation with the CA, the protein band of the modified antibody was shifted toward a higher molecular weight, indicating almost complete interaction with the CA (Figure 4B).

Initial in vivo CA studies were performed with non-radiolabeled TCO-modified antibodies, as iodination is dependent on oxidation with chloramine-T, which could damage the TCO group and disturb interaction with the CA. Thus, TCO-RmAb158-scFv8D3 or TCO-RmAb158 was injected in wt mice, followed by CA administration at 1 or 72 h after antibody injection. As seen in Figure 4C, injection of the CA had some effect on the plasma concentration of TCO-RmAb158-scFv8D3, with approximately half of the antibody concentration remaining at 24 h after antibody injection, when compared with animals that did not receive CA. In contrast, CA administration reduced plasma concentration of TCO-RmAb158 substantially and immediately, when administered at both 1 and 72 h after antibody injection (Figure 4D).

To study why the bispecific RmAb158-scFv8D3 was largely unaffected by both strategies to enhance blood clearance, an additional experiment was performed, hypothesizing that the effect of both clearing strategies could be dependent on the fraction of free antibody in plasma. Thus, [^125^I]I-RmAb158-scFv8D3 and [^125^I]I-RmAb158 were injected in tg-ArcSwe mice and the amount of radioactivity in plasma was compared with that in total blood and in blood cell pellet. This experiment showed that only 10–20% of [^125^I]I-RmAb158-scFv8D3 was free in plasma and the rest was bound to blood cells, while [^125^I]I-RmAb158 was more available in plasma, with around 90% found in the plasma fraction (Figure 4E).

Because clearance of RmAb158-scFv8D3 was less efficient, further studies, including SPECT imaging, were done with RmAb158. This antibody has previously been shown to accumulate specifically around the brain’s ventricles, bound to Aβ deposits. This specific retention was abundant 3 days after antibody injection, but due to high antibody concentration in blood, it was not readily visualized with SPECT until 6–14 days after administration.^28^ Induced clearance of the antibody could therefore improve [^125^I]I-RmAb158 SPECT imaging. To enable the combination of TCO-modification and radiolabeling, which is a prerequisite for SPECT imaging, iodination of the antibody was performed before TCO-modification, as reasoned above.

**Figure 5:**
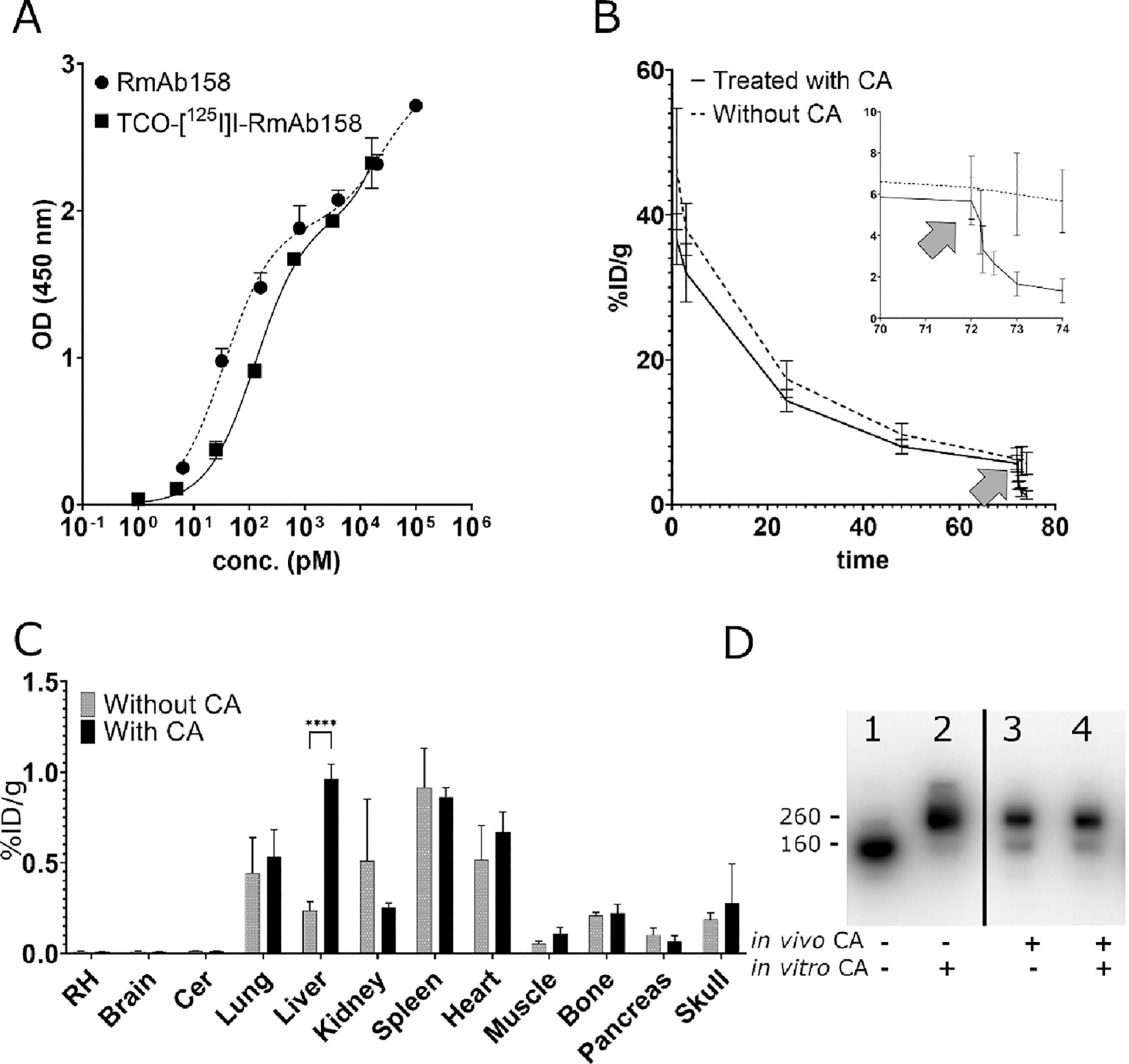
(A) ELISA analysis of RmAb158 binding to Aβ protofibrils before and after iodination and TCO-modification (B). Blood pharmacokinetics of [^125^I]I-RmAb158 over time, with CA administration 72 h after antibody injection (indicated by gray arrow). Inset shows magnification of the last 4 h. (C) Biodistribution of TCO-[^125^I]I-RmAb158 D. Autoradiograph of SDS-PAGE gel loaded with plasma from TCO-[^125^I]I-RmAb158 injected mice without CA treatment (1, 2) or after in vivo administration of CA (3, 4). In 2 and 4, CA was added to plasma in vitro. Data are represented in mean ± standard deviation. Significant differences between groups were tested with two-way ANOVA, followed by Bonferroni’s *post hoc* test (C), (****p < 0.0001).

To assess CA-induced blood clearance of RmAb158 that was both radiolabeled and TCO-modified, the antibody was injected in wt mice. Three days after injection of TCO-[^125^I]I-RmAb158, the CA was injected in half of the mice. The radioactivity signal in blood dropped immediately after injection of the CA (Figure 5B). In another experiment, mice euthanized 1.5 h after CA administration displayed a considerably higher radioactive signal in the liver compared to non-injected animals. This is in line with the expected mechanism of action (Figure 5C). CA interaction with TCO-[^125^I]I-RmAb158 was also assessed ex vivo in plasma samples from mice injected with TCO-[^125^I]I-RmAb158, before or after CA administration. In plasma taken before CA administration, a shift in band size was observed after in vitro addition of an excess of CA (Figure 5D, lane 1–2). In plasma taken after CA administration, bands were already shifted due to in vivo interaction with CA and were not further changed by in vitro addition of CA (Figure 5D, lane 3–4). Quantification of the bands showed 77% efficiency of CA in clearing TCO-[^125^I]I-RmAb158 from plasma in vivo.

**Figure 6:**
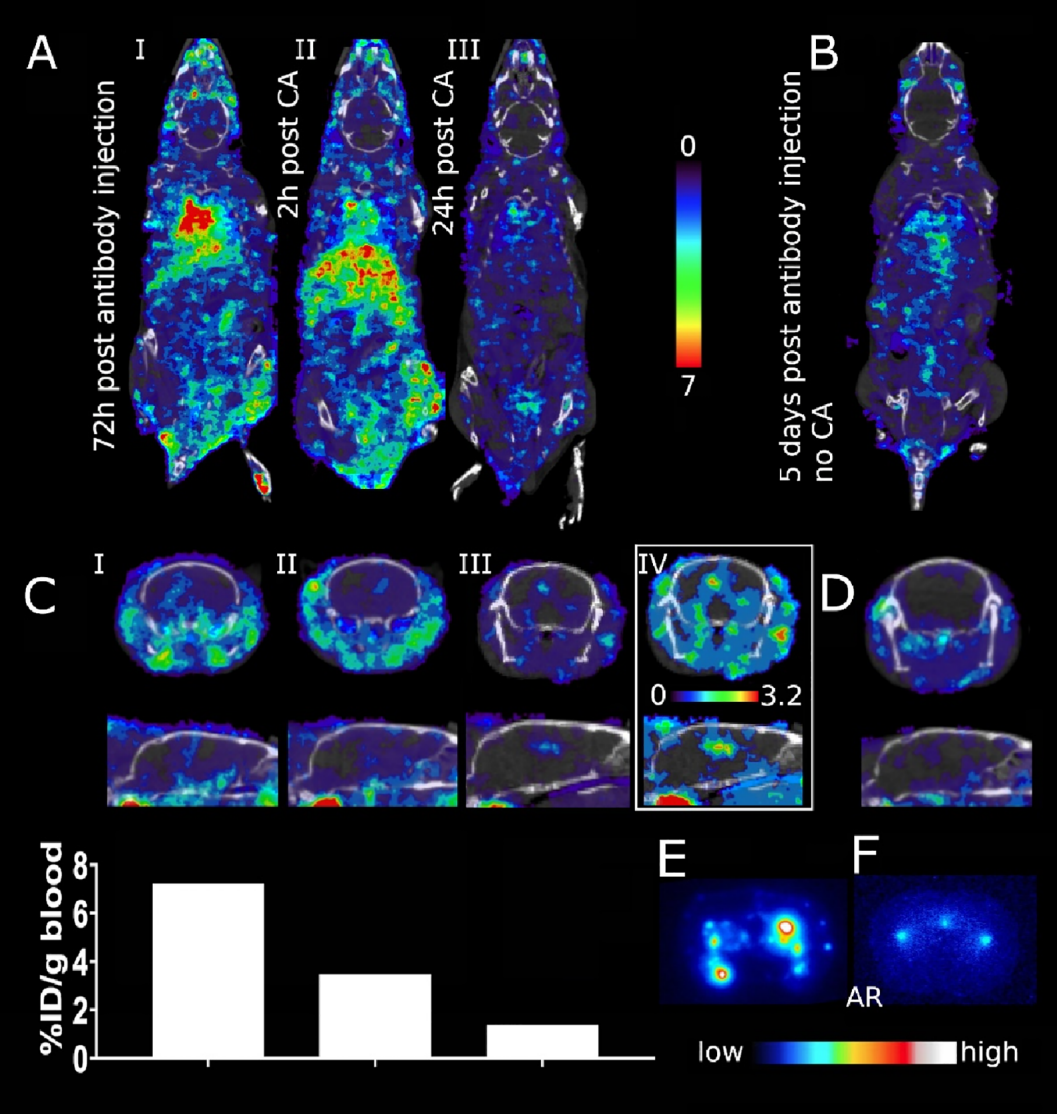
(A) Whole body SPECT scans of 18–20 month old tg-ArcSwe mouse injected with TCO-[^125^I]I-RmAb158 72 h before the first scan. Images, scaled to the same maximum threshold of an arbitrary unit for comparability, show radioactive signals, before (I), 2 h after (II), and 24 h after (III) CA administration. (B) Non-CA-injected tg-ArcSwe mouse 5 days post injection of TCO-[^125^I]I-RmAb158. (C) CA impact on brain imaging of ventricular TCO-[^125^I]I-RmAb158 retention in same mice as (A). TCO-[^125^I]I-RmAb158 retention was barely visible after 3 days (I). After CA administration (II), blood contained less radiolabeled antibody and imaging of ventricular TCO-[^125^I]I-RmAb158 accumulation could be visualized, with further increased contrast 24 h after CA administration (III and IV; in IV, the maximum image threshold was decreased). Graph underneath represents antibody concentration in blood (% ID/g) at time points corresponding to SPECT images I–III. (D) Brain image of TCO-[^125^I]I-RmAb158 injected wt mouse, 24 h after CA administration, showing no specific brain retention of TCO-[^125^I]I-RmAb158. Ex vivo autoradiography images of brain tissue from tg-ArcSwe (E) and wt (F) mice.

Finally, to investigate the impact of the induced clearance on SPECT imaging, TCO-[^125^I]I-RmAb158 was injected in 18–20 month old tg-ArcSwe and wt mice and scanned at three different time points—before, 2 h after and 24 h after injection of the CA (Figure 6A). As seen in the SPECT images, the signal in the liver increased substantially after CA injection and resulted in an almost complete clearance of the antibody from the body after 24 h (Figure 6AI–III). In comparison, a mouse scanned 5 days after TCO-[^125^I]I-RmAb158 injection showed a higher overall signal of radioactivity in the body (Figure 6B). Clearance of TCO-[^125^I]I-RmAb158 from the blood allowed for the visualization of ventricle-associated brain signal from the retained antibody (Figure 6CIII,IV). Sagittal and coronal images of the brain showed a distinct signal in the center of the brain, apparent at 2 h and especially at 24 h after CA administration (Figure 6CII–IV), clearly related to the decreased antibody blood concentration (Figure 6C). This signal likely represents accumulation of the antibody in association with Aβ deposits around the central ventricle, a phenomenon that has previously been observed with SPECT imaging 6–27 days after the injection of [^125^I]I-RmAb158 in tg-ArcSwe mice.^28^ No signal was observed in the brain of TCO-[^125^I]I-RmAb158 injected wt mice 24 h after CA administration (Figure 6D). The specificity of the SPECT signal was confirmed by ex vivo autoradiography, showing high antibody retention in the tissue of tg-ArcSwe (Figure 6E) but not wt (Figure 6F) mice in the absence of blood.

**Figure 7:**
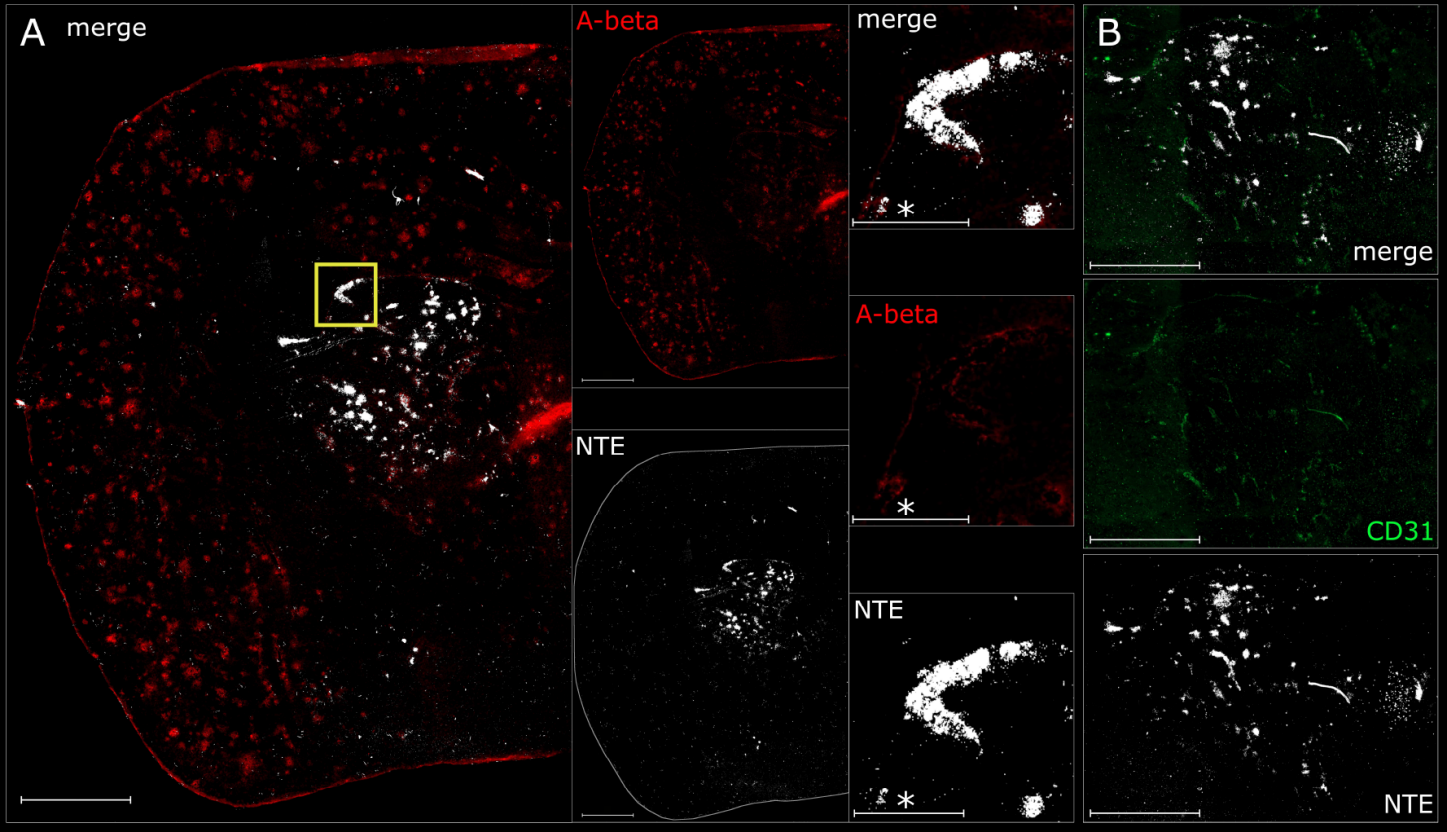
Nuclear track emulsion microautoradiography from *post-mortem* coronal cryosectioned brain of a CA-treated and SPECT-scanned tg-ArcSwe mouse. Scale bar = 1000 μm, *scale bar = 250 μm. NTE detection of TCO-[^125^I]I-RmAb158 (white puncta) in combination with immunostaining of (A) Aβ (red), close-up area highlighted in yellow, and (B) endothelial cell marker CD31 (green).

To image the TCO-[^125^I]I-RmAb158 brain distribution at a higher resolution, nuclear track emulsion microautoradiography combined with Aβ and endothelial immunofluorescence staining was performed. This analysis visualized the interaction of the radiolabeled antibody with Aβ plaques and the brain vasculature (Figure 7A). In line with the SPECT-images, TCO-[^125^I]I-RmAb158 showed defined hot-spots in the lateral ventricle as well as in thalamic vessels, which co-localized with Aβ-pathology (Figure 7B).

## Discussion

To achieve high contrast in molecular imaging with radioligands that have a long residence time in blood is challenging. Hence, specific strategies are being developed to clear excess radioligand from the blood circulation. Such strategies have mainly been applied in imaging of peripheral targets, such as tumors. However, with emerging attempts to use antibody-based imaging of brain targets, strategies for increased blood clearance need to be developed. Here, to obtain distinct images of the AD related Aβ pathology in a preclinical research setting, the Aβ protofibril binding antibody RmAb158 and its brain penetrating bispecific variant, RmAb158-scFv8D3, were investigated. To increase the peripheral clearance for both antibody variants, we used two different types of modifications—direct mannosylation of the antibodies and TCO-functionalization in combination with a tetrazine-functionalized albumin-based CA.

Mannosylation of an antibody has previously been used to image myocarditis.^41^ This approach was successful, as the target was easily accessible from the blood stream. To assess the effect of a mannosylated antibody on brain imaging, we investigated its concentration in blood over 24 h after antibody injection. The data indicated a rapid clearance of mannose-[^125^I]I-RmAb158 via the liver.^16^ However, due to the fast clearance from blood, a corresponding reduction in brain delivery was also observed. Thus, the beneficial effect of clearance was counteracted by its effect on the brain concentration. In summary, the findings demonstrated the possibility of using binding to a peripheral target to modulate antibody circulation time as well as the importance of matching the kinetics between clearance and target tissue uptake.

Instead, an inducible clearance strategy was employed, allowing a TCO-functionalized antibody to circulate for a certain amount of time, to accumulate at the brain target, before clearance of the peripheral signal from blood was induced with a previously published tetrazine-based CA.^19^ This CA was designed to target the Ashwell receptor, which is expressed by hepatic parenchymal cells,^43,44^ and has been shown to rapidly clear from the blood.^45^ This strategy uses the IEDDA reaction to achieve a fast click reaction between the antibody and CA in the blood. TCO is prone to isomerize in vivo, making it difficult to use,^46^ but isomerization can be overcome by conjugating TCO groups to an antibody, which provides a microenvironment that protects the TCO from isomerization and prolongs the TCO reactivity in vivo. The IEDDA reaction is rapid and potent, and with the large molar excess of tetrazine over TCO groups in the present study, clearance of the TCO-functionalized antibody is expected to be effective. Indeed, blood pharmacokinetic experiments confirmed the fast interaction of the CA with the TCO-modified antibody, leading to immediate clearance of TCO-[^125^I]I-RmAb158 from the blood, in line with previous studies.^46,47^ Furthermore, experiments demonstrated that the TCO-group on the circulating antibody was reactive in vivo up to 3 days after injection.

Previous studies have shown that the fusion of a TfR binder (8D3) to anti-Aβ antibodies substantially increased their brain uptake and improved their spatial distribution within the brain.^25,29,31,39,48−50^ These features enabled their use as PET radioligands for brain-imaging. Antibody-based imaging studies have been conducted mainly using long-lived radionuclides such as iodine-124, which allows for imaging at a late time point, usually several days after administration of the antibody-based radioligand.^27,51^ However, this combination of antibody and radionuclide, due to their long half-lives, would lead to high exposure of radiation to patients and is therefore not suitable in a clinical setting. Instead, short-lived radionuclides, such as fluorine-18, are desirable for clinical use and could reduce the dose of radioactivity to the patient. Our previous attempts to carry out brain PET imaging with ^18^F-labeled antibodies clearly indicated that the long biological half-life of the antibodies did not match the rapid decay of fluorine-18,^30^ suggesting that an approach for increased clearance could be beneficial. However, attempts to enhance the contrast of RmAb158-scFv8D3, either with direct mannose modification, or using peripherally administered CA, did not substantially affect blood clearance of RmAb158-scFv8D3. Further investigation showed that only 10–20% of RmAb158-scFv8D3 was freely available in plasma. The majority of the bispecific antibody was likely bound to TfR, which is abundantly expressed on blood cells.^52^ Studies have shown that bispecific antibodies displaying bivalent TfR interactions might cluster TfR at the cell surface, which could result in low plasma availability^39,53^ and poor clearance from blood. RmAb158-scFv8D3 has two TfR binding moieties with relatively high TfR affinity, which promotes a strong and potentially bivalent TfR binding. With an antibody construct that favors monovalent TfR binding of lower affinity, the free fraction in plasma would likely be higher, suggesting it could be worth to revisit the induced clearance strategy with such antibody constructs.

Even though the clearance strategies explored here were not compatible with bispecific antibodies interacting with TfR, the proof-of-concept was demonstrated with RmAb158. Previous studies have shown that the concentration of RmAb158 in the brain peaks 3 days post injection^26,28^ but the blood concentration is still high. Additionally, a long-term SPECT study showed a specific retention of RmAb158 in association with Aβ deposits around ventricles in the brain of tg-ArcSwe mice up to 27 days after injection.^28^ Inspired by these studies, we decided on a 3 day circulation time of the antibody in vivo before injection of the CA. After administration of the CA, an increased signal immediately appeared in the liver, which aligns with previous observations,^19^ and a specific, centrally located signal gradually appeared in the brain, clearly observed by SPECT-imaging. The signal location and specificity to Aβ was confirmed by ex vivo autoradiography and nuclear track emulsion microautoradiography *post mortem*. Furthermore, wt mice, which were treated similarly as tg-ArcSwe mice, showed low brain signals, confirming that the specific retention of the antibody in the brain was associated with Aβ pathology. As RmAb158 was not equipped with a TfR-binder and therefore not able to cross the BBB via RMT, it is assumed that the long blood half-life, combined with highly perfused and permeable vasculature in the blood–cerebrospinal fluid barrier results in a local influx of antibodies and a very specific pattern of ventricular retention.^54^

In conclusion, this study demonstrates that the principle of induced radioligand clearance, based on the biorthogonal IEDDA reaction, can be used for antibody-based imaging in the CNS. With further optimization of antibody design and radiolabeling, this may become a useful strategy to enhance contrast in antibody-based imaging of brain targets.
